# Heat-killed *Lactobacillus casei* confers broad protection against influenza A virus primary infection and develops heterosubtypic immunity against future secondary infection

**DOI:** 10.1038/s41598-017-17487-8

**Published:** 2017-12-12

**Authors:** Yu-Jin Jung, Young-Tae Lee, Vu Le Ngo, Young-Hee Cho, Eun-Ju Ko, Sung-Moon Hong, Ki-Hye Kim, Ji-Hun Jang, Joon-Suk Oh, Min-Kyung Park, Cheol-Hyun Kim, Jun Sun, Sang-Moo Kang

**Affiliations:** 1 Center for Inflammation, Immunity & Infection, Institute for Biomedical Sciences, Georgia State University, Atlanta, Georgia 30303 USA; 2 Department of Animal Resource Science, Dankook University, 119, Dandae-ro, Dongnam-gu, Cheonan-si, Chungnam 330-714 Korea; 3 Tobico Inc. Chungnam Techno Park, Jiksan-Eup, Seobuk-Gu, Cheonan-Si, Chungnam 331-858 Korea; 4 Department of Human Nutrition and Food Science, Chungwoon University, Namjang-Ri, Hongsung-Eup, Hongsung-Kun, Chungnam 350-701 Korea; 5 Division of Gastroenterology and Hepatology, Department of Medicine, University of Illinois at Chicago, Chicago, USA

## Abstract

Lactic acid bacteria (LAB) are the common probiotics. Here, we investigated the antiviral protective effects of heat-killed LAB strain *Lactobacillus casei* DK128 (DK128) on influenza viruses. Intranasal treatment of mice with DK128 conferred protection against different subtypes of influenza viruses by lessening weight loss and lowering viral loads. Protection via heat-killed DK128 was correlated with an increase in alveolar macrophage cells in the lungs and airways, early induction of virus specific antibodies, reduced levels of pro-inflammatory cytokines and innate immune cells. Importantly, the mice that were protected against primary viral infection as a result of heat-killed DK128 pretreatment developed subsequent heterosubtypic immunity against secondary virus infection. For protection against influenza virus via heat-killed DK128 pretreatment, B cells and partially CD4 T cells but not CD8 T cells were required as inferred from studies using knockout mouse models. Our study provides insight into how hosts can be equipped with innate and adaptive immunity via heat-killed DK128 treatment to protect against influenza virus, supporting that heat-killed LAB may be developed as anti-virus probiotics.

## Introduction

Influenza virus can cause serious respiratory disease in humans. Despite the availability of influenza vaccines, it is estimated that influenza virus infections cause 3 to 5 million severe illnesses and 250,000 to 500,000 influenza-related deaths worldwide during epidemics^1–3^. Current influenza vaccines are effective when vaccine strains are well matched with the circulating influenza viruses. A recent outbreak of the new 2009 H1N1 pandemic virus represents an example of the limited efficacy of the current vaccination^4,5^. Influenza A virus infects various hosts including humans, birds, and pigs. A variety of influenza A viruses are present in many different subtypes based on hemagglutinin (HA) and neuraminidase (NA) proteins on the surface of the virus. At present, 18 different HA (H1-H18) and 11 different NA (N1-N11) subtype molecules are identified, indicating the existence of numerous HA and NA combinations of influenza viruses^6^. Therefore, it is important to find an alternative measure that would provide protection against influenza virus regardless of strain-specificity. Lactic acid bacteria (LAB) are the most common probiotics which bestow health benefits on the host as micro-organisms. Various fermented vegetables and dairy products contain a variety of LAB, which were shown to provide health benefits^1,7–10^.

Some LAB strains as probiotics were reported to partially protect against bacterial infectious diseases, such as *Streptococcus pyogenes* and *Streptococcus pneumoniae* ^11,12^. Also, a variety of LAB strains were shown to protect mice against influenza virus infections by improving survival rates after intranasal or oral pretreatment but not preventing severe disease of weight loss^13,14^. Yogurt fermented with *Lactobacillus* was shown to reduce the cases of catching cold in the healthy elderly^15^ and to prolong the survival periods of mice with influenza virus infection^16^. In particular, previous studies have demonstrated the protective effects against influenza virus infection by administration of various LAB strains via the oral route^3,17–20^ or the intranasal route^14,20–24^. However, in the previous studies, the probiotic effects of LAB on influenza virus infection include partial protection or prolonged survival periods, accompanying substantial weight loss and resulting in various efficacy depending on the strains and routes of LAB.

It remains unknown whether pretreatments with heat-killed LAB can confer protection by preventing weight loss of animals after influenza virus infection and thus ameliorating morbidity. Furthermore, the antiviral protective mechanisms by LAB are poorly understood. In the present study, we found that heat-killed *Lactobacillus casei* strain DK128 treatment of mice conferred strain-nonspecific protection against morbidity of weight loss and mortality due to lethal influenza virus infection. Infection permissive protection against primary viral infection via heat-killed DK128 pretreatments was found to equip the mice with cross-protective immunity against secondary lethal infection with a heterosubtypic virus. The possible underlying mechanisms of the antiviral effects of DK128 were investigated.

## Results

### Intranasal pretreatments with heat-killed DK128 confers protection against influenza H3N2 virus

**Figure 1:**
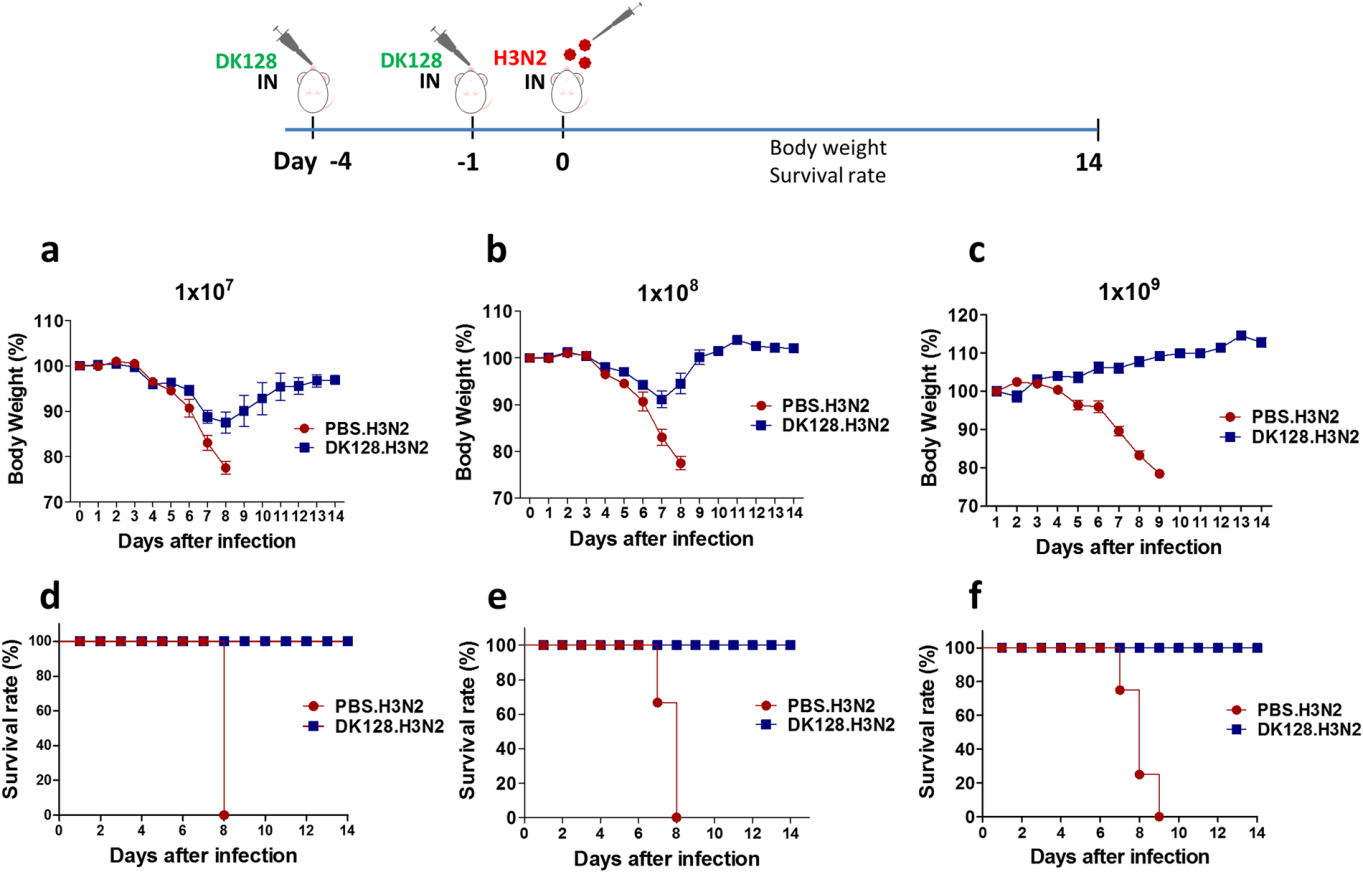
Pretreatment of mice with heat-killed DK128 confers protection against H3N2 influenza virus (A/Phil/82) infection. BALB/c mice (n = 5/group) were intranasally (IN) pretreated with different doses of heat-killed DK128 at 4-day and 1-day prior to H3N2 influenza virus (2 LD_50_, A/Philippines/2/1982) infection. (**a**) Body weight change after H3N2 virus infection of mice with heat-killed DK128 (1 × 10^7^ CFU/50 ul/mouse) pretreatment. (**b**) Body weight change after H3N2 virus infection of mice with heat-killed DK128 (1 × 10^8^ CFU/50 ul/mouse) pretreatment. (**c**) Body weight change after H3N2 virus infection of mice with heat-killed DK128 (1 × 10^9^ CFU/50 ul/mouse) pretreatment. (**d**) Survival rates after H3N2 virus infection of mice with heat-killed DK128 (1 × 10^7^ CFU/50 ul/mouse) pretreatment. (**e**) Survival rate after H3N2 virus infection of mice with heat-killed DK128 (1 × 10^8^ CFU/50 ul/mouse) pretreatment. (**f**) Survival rate after H3N2 virus infection of mice with heat-killed DK128 (1 × 10^9^ CFU/50 ul/mouse) pretreatment. PBS.H3N2: mock-treated mice infected with H3N2 influenza virus. DK128.H3N2: mice with heat-killed DK128 pretreatment prior to H3N2 virus infection.

In our previous study, we have reported that intranasal pretreatments with live *Lactobacillus plantarum* DK119 could develop resistance to influenza virus H1N1 infection in mice despite a certain degree of morbidity^20^. LAB DK128, a new isolate from fermented vegetables, was suggested to be a promising probiotic^25^. To determine whether pretreatments with heat-killed LAB endows mice with resistance to influenza virus, mock and heat-killed DK128-treated mice (BALB/c) were infected with a lethal dose of A/Philippines/82 (H3N2) virus (Fig. 1). BALB/c mice that were treated with heat-killed DK128 at a low dose, 1 × 10^7^ CFU or 1 × 10^8^ CFU showed approximately 12% to 10% weight loss (Fig. 1a,b) but all survived the lethal infection with H3N2 virus. In contrast, mice treated with heat-killed DK128 at a higher dose (1 × 10^9^ CFU) prior to infection did not show weight loss, whereas mock-treated mice displayed severe weight loss reaching to the endpoint by day 8–9 post infection and all died (Fig. 1d,e,f). Thus, the efficacy of protection against influenza virus via heat-killed DK128 appeared to be dependent on the LAB doses of pretreatments. These results are highly significant because mice with heat-killed DK128 pretreatments can be protected against lethal influenza virus infection, resulting in 100% survival and prevention of weight loss.

### Mice with heat-killed DK128 pretreatment control lung viral loads and virus-induced pro-inflammatory cytokines to lower levels

**Figure 2:**
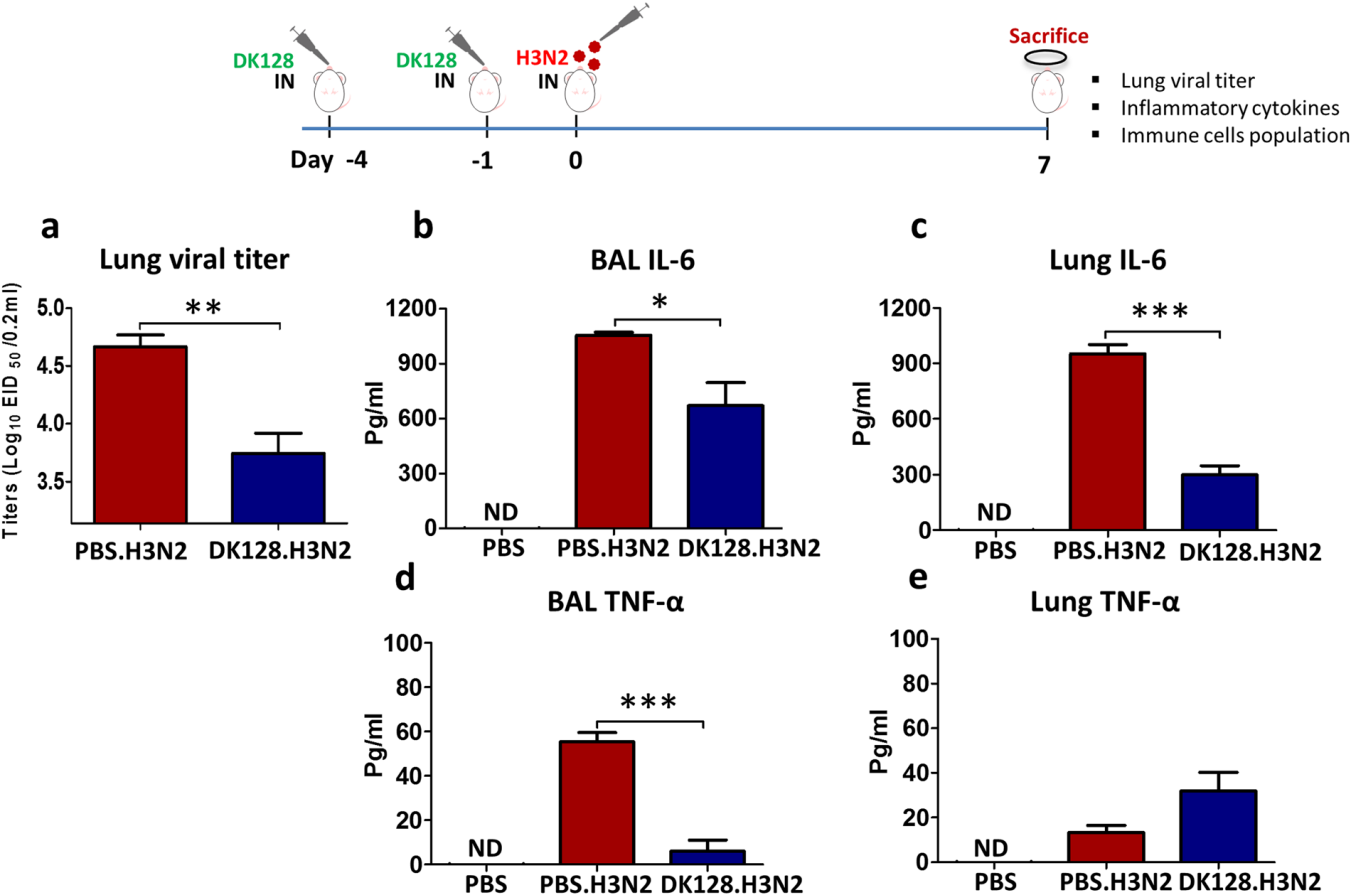
Pretreatment of BALB/c mice with heat-killed DK128 lowers lung viral loads and inflammatory cytokines upon H3N2 influenza virus infection. BALB/c mice (n = 5/group) were intranasally (IN) pre-treated with heat-killed DK128 (1 × 10^9^ CFU/50 μl/mouse) at 4-day and 1-day prior to infection with H3N2 virus (2 LD_50_, A/Philippines/2/1982). Mice were sacrificed at 7 days after infection to determine lung virus titers and cytokines in bronchoalveolar lavage fluids (BALF) and lung extracts. (**a**) Lung virus titers (Log_10_ EID_50_ /0.2 ml). (**b**) IL-6 (pg/ml) in BALF. (**c**) IL-6 (pg/ml) in lung. (**d**) TNF-α (pg/ml) in BALF. (**e**) TNF-α (pg/ml) in lung. PBS: uninfected mouse control. PBS.H3N2: mock-treated mice infected with H3N2 influenza virus. DK128.H3N2: heat-killed DK128 pretreatment prior to H3N2 virus infection. ND indicates not detected. Cytokine levels were described as mean ± SEM. Symbols *, *** denote p < 0.05 and 0.001 respectively by Two-tailed Student’s paired *t* test. The viral loads in the lungs was described as mean ± SEM. ** denotes p < 0.01 by Two-tailed Student’s paired *t* test.

In an independent set of experiments, we determined the efficacy of clearing lung viral loads, which is an important parameter for assessing protective efficacy. At 7 days after infection, mice were sacrificed and lung extract samples were diluted to determine egg infectious titers (Fig. 2a). Heat-killed DK128 (10^9^ CFU) treated mice showed approximately 18-fold lower levels of viral titers than those in mock-treated mice after infection. Infection with a pathogenic influenza virus can cause excessive production of proinflammatory cytokines. IL-6 and TNF-α inflammatory cytokines were determined in BALF and lung samples. At 7 days after infection, IL-6 was detected at significantly lower levels in BALF and lung samples from heat-killed DK128 pretreated mice than those in mock-treated mice after infection (Fig. 2b,c). TNF-α was also significantly lower in BALF from heat-killed DK128 pretreated mice compared to naïve mice (Fig. 2d) despite no statistical difference in the lung TNF-α levels between the two groups (Fig. 2e). Therefore, the induction of proinflammatory cytokines due to viral infection was substantially reduced as a result of heat-killed DK128 treatment.

### Recruitment of virus-induced innate immune cells is differentially modulated by heat-killed DK128 pretreatment

**Figure 3:**
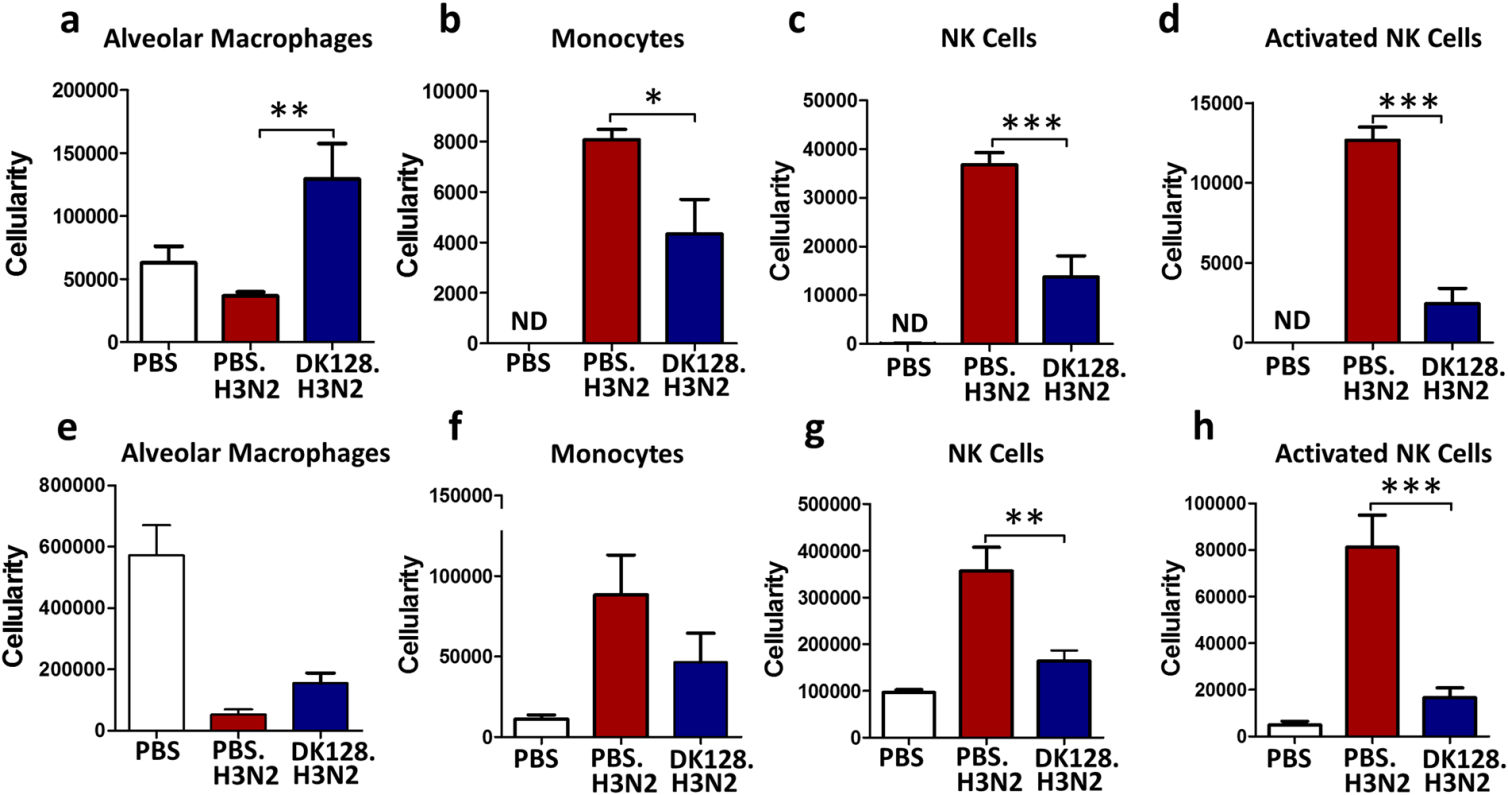
BALB/c mice with heat-killed DK128 pretreatment differentially modulate infiltrating innate immune cells in bronchoalveolar lavage fluids (BALF) and lungs upon H3N2 influenza virus infection. BALB/c mice (n = 5/group) were intranasally (IN) pretreated with heat-killed DK128 (1 × 10^9^ CFU/50 μl/mouse) at 4-day and 1-day prior to H3N2 virus (2 LD_50_, A/Philippines/2/1982) infection. Mice were sacrificed day 7 post-infection to determine cell phenotypes and cellularity of infiltrating immune cells in BALF and lungs. (**a**–**d**) Infiltrated immune cells in BALF (cellularity/mouse); (**a**) Alveolar macrophages (CD11c^+^CD11b^−^F4/80^+^), (**b**) Monocytes (CD11c^−^CD11b^+^siglecF-Ly6c^hi^F4/80^+^), (**c**) NK cells (F4/80-DX5^+^), (**d**) Activated NK cells (F4/80^−^DX5^+^CD69^+^). (**e**–**h**) Infiltrating immune cells in the lungs (cellularity/mouse); (**e**) Alveolar macrophages (CD11c^+^CD11b^−^F4/80^+^), (**f**) Monocytes (CD11c^−^CD11b^+^siglecF-Ly6c^hi^F4/80^+^), (**g**) NK cells (F4/80-DX5^+^), (**h**) Activated NK cells (F4/80^−^DX5^+^CD69^+^). PBS: uninfected mouse control. PBS.H3N2: mock-treated mice infected with H3N2 influenza virus. DK128.H3N2: heat-killed DK128 pretreatment prior to H3N2 virus infection. The numbers of immune cells (per mouse in BALF or in the lung) were described as mean ± SEM. *, **, and *** denote p < 0.05, 0.1, and 0.001 respectively by One-way ANOVA and Tukey’s multiple comparison test.

Pathogenic influenza virus can cause severe inflammatory disease in lower respiratory tracts and lungs. Thus, the analysis of immune cell populations recruited to the site of virus infection would provide insight into the possible protective mechanisms mediated by heat-killed DK128 pretreatment. At 7 days after infection with lethal A/Philippines (H3N2) virus, we collected lung and BALF samples to analyze phenotypes of immune cells by multi-color flow cytometry. Alveolar macrophage phenotypic (CD11C^+^CD11b^−^F4/80^+^) cells were observed at higher levels in BALF from mice with heat-killed DK128 pretreatment than those in mock-treated mice with severe weight loss (Fig. 3a).

In contrast, BALF and lung samples from mice with heat-killed DK128 pretreatment showed lower levels of monocytes (F4/80^+^CD11c^−^CD11b^+^Ly6C^hi^SiglecF^−^), natural killer cells (F4/80^−^DX5^+^CD69^+^), and activated natural killer cells (F4/80^−^DX5^+^CD69^+^) (Fig. 3b–d,f–h), while naïve untreated-mice showed significantly enhanced recruitment of monocytes, natural killer cells, and activated natural killer cells in response to the lethal infection with A/Philippines (H3N2) virus (Fig. 3b–d,f–h). Cellular analysis of BALF and lungs suggest that LAB DK128-mediated modulation of innate immune cells may prevent pulmonary inflammation, contributing to protection against influenza virus infection.

To further detail the possible role of alveolar macrophages, we applied clodronate-liposome to deplete airway macrophages in mice with heat-killed LAB treatment prior to influenza virus lethal infection (Supplementary Fig. 1). We confirmed that over 90% alveolar macrophages were depleted in the lungs after clodronate-liposome treatment prior to influenza virus infection (Supplementary Fig. 1a). Upon lethal infection with influenza virus, the clodronate-treated LAB DK128 mice displayed severe weight loss of over 20% and a survival rate of 25% whereas control LAB DK128 mice (100%) survived lethal infection and prevented weight loss (DK.H3N2, Supplementary Fig. 1b,c). All mice without heat-killed LAB treatment died of infection (data not shown). These data indicate the protective roles of alveolar macrophages that were elevated by prior treatment with heat-killed LAB.

### Heat-killed DK128 pretreatment of mice mediates earlier induction of virus specific antibodies upon infection

**Figure 4:**
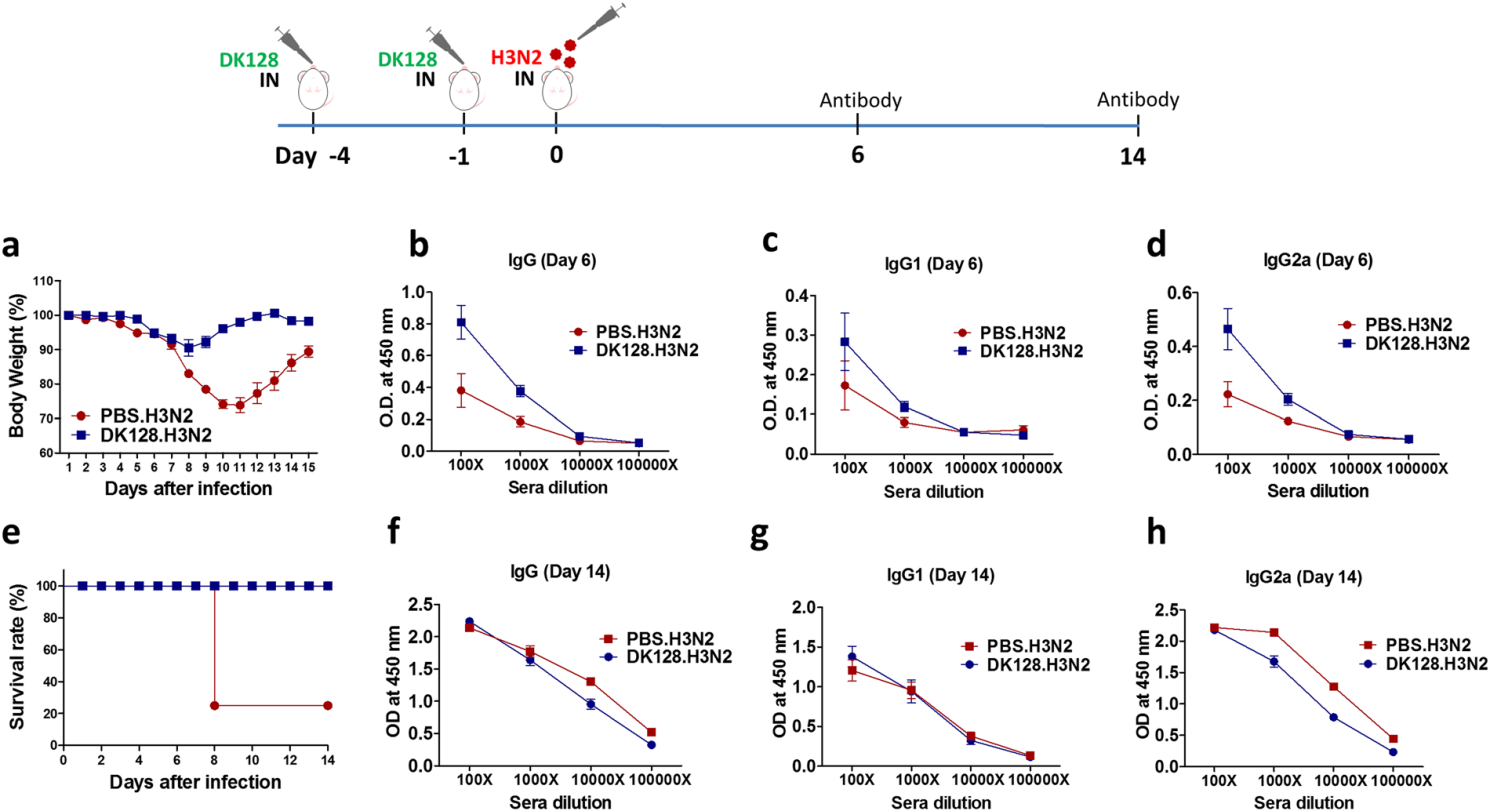
Heat-killed DK128 pretreatment mediates early induction of IgG antibodies after H3N2 virus infection. BALB/c mice (n = 5/group) were intranasally (IN) pretreated with heat-killed DK128 (1 × 10^8^ CFU/50 μl/mouse) at 4-day and 1-day prior to infection with H3N2 virus (1.5 LD_50_, A/Philippines/2/1982). At 6 and 14 days after H3N2 virus infection, serum was collected and the levels of IgG and IgG isotypes specific for H3N2 virus were measured by enzyme-linked immunosorbent assay (ELISA). (**a**) Body weight changes in mice after H3N2 virus infection. (**b**–**d**) Antibody responses at 6 days after H3N2 virus infection; (**b**) IgG, (**c**) IgG1, (**d**) IgG2a. (**e**) Survival rates of mice after H3N2 virus infection. (**f**–**h**) Antibody responses at 14 days after H3N2 virus infection; (**e**) IgG, (**f**) IgG1, (**g**) IgG2a. PBS.H3N2: untreated mice with H3N2 virus infection, DK128.H3N2: mice with heat-killed DK128 pretreatment prior to H3N2 virus infection.

B cells are responsible for producing antibodies that play a critical role in establishing long-lived adaptive immunity. To better understand the possible roles of antibodies in conferring protection in the heat-killed DK128 pretreated mice, we compared virus specific antibody responses in heat-killed DK128 (10^8^ CFU) treated and untreated BALB/c mice at 6 days after infection of with a low dose (1.5 x LD_50_) of H3N2 virus (Fig. 4). Heat-killed DK128 pretreated mice displayed a moderate level of weight loss (6–9%) whereas mock control mice exhibited severe weight loss (20–25%) resulting in partial survival rates upon H3N2 virus infection (Fig. 4a,e). Interestingly, the mice with heat-killed DK128 pretreatment raised significantly higher levels of IgG, IgG1, and IgG2a antibodies at an earlier time point of day 6 post infection compared to those in mock untreated mice with infection (Fig. 4b,c,d). At a later time point of 14 days after infection, H3N2 virus infected naïve untreated mice that exhibited severe weight loss reached the high levels of IgG, IgG1, and IgG2a antibodies comparable to those from heat-killed DK128 treated mice (Fig. 4f,g,h). These results suggest that mice with heat-killed DK128 pretreatment are effective in inducing early IgG antibodies, possibly contributing to controlling viral replication as well as reducing weight loss whereas the untreated mice are ineffective in producing IgG antibodies at early times post infection, resulting in high viral replication and severe weight loss, and likely to die.

### Protected BALB/c mice against H3N2 virus primary infection by heat-killed DK128 pretreatment acquire heterosubtypic immunity against secondary infection later

**Figure 5:**
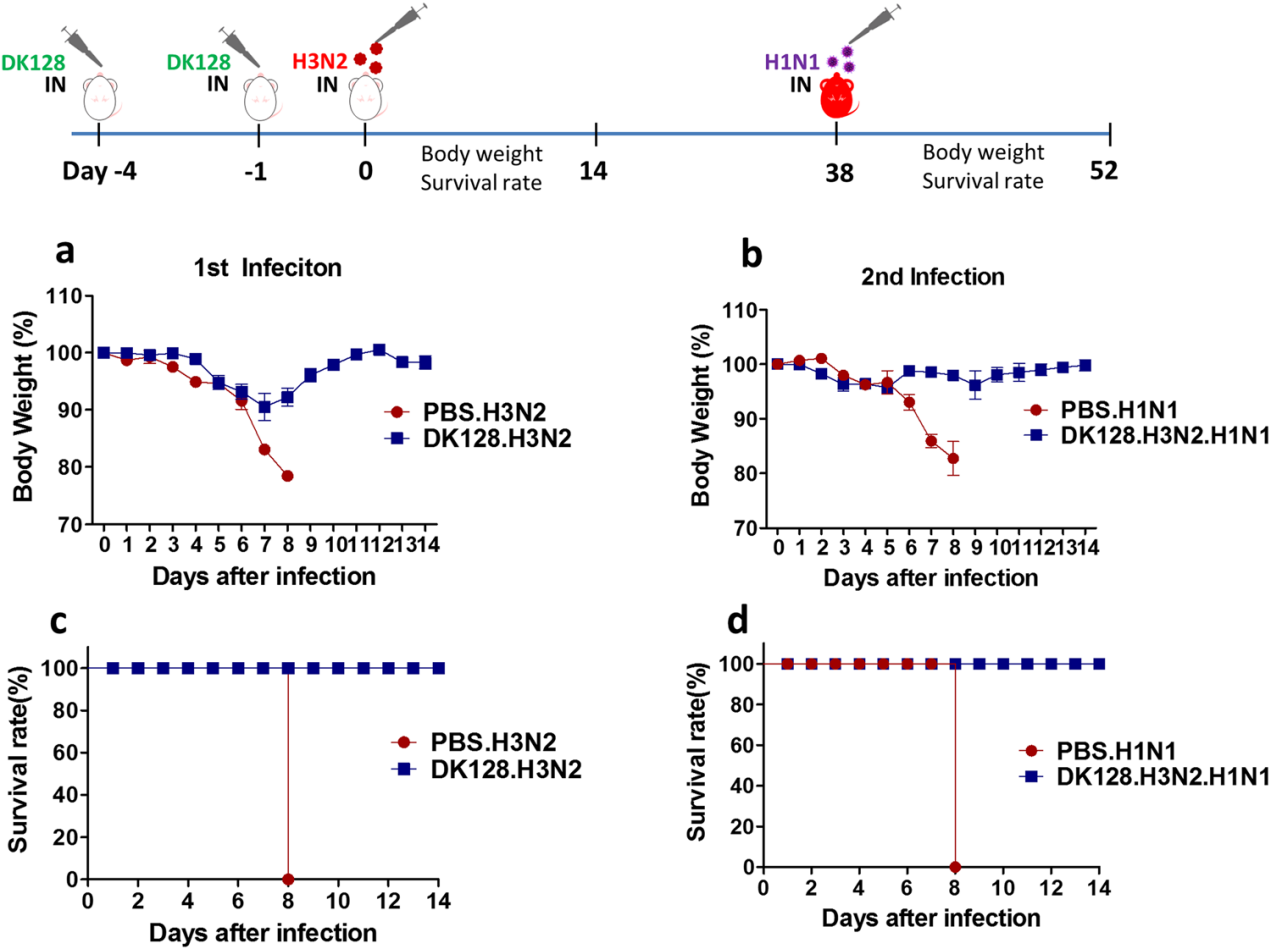
Mice with heat-killed DK128 mediated protection against H3N2 virus primary infection show immunity to heterosubtypic H1N1 virus secondary infection. BALB/c mice (n = 5/group) were intranasally (IN) pretreated with heat-killed DK128 (10^9^ CFU/50 μl/mouse) at 4-day and 1-day prior to infection with H3N2 virus (2 LD_50_, A/Philippines/2/1982). Body weight change and survival rate were monitored for 14 days. After primary virus (H3N2) infection, the protected mice were secondary infected with heterosubtypic virus (H1N1, 5 LD_50_, A/California/04/2009) and then weight loss and survival rate were monitored for 14 days. (**a**) Body weight changes after primary infection of BALB/c mice with H3N2 virus. (**b**) Body weight changes after secondary infection of BALB/c mice with H1N1 virus. (**c**) Survival rates after primary infection of BALB/c mice with H3N2 virus. (**d**) Survival rates after secondary infection of BALB/c mice with H1N1 virus. PBS.H3N2: H3N2 virus infection, DK128.H3N2: mice with heat-killed DK128 pretreatment prior to H3N2 virus infection, PBS.H1N1: H1N1 virus infection, DK128.H3N2.H1N1: The protected-mice against primary H3N2 virus via heat-killed DK128 pretreatment were then infected with secondary H1N1 virus.

It is highly desirable for the hosts to acquire immunity against primary and secondary heterosubtypic viral infections without morbidity (weight loss). To determine cross-protective immunity against subsequent secondary infection, BALB/c mice that were protected against H3N2 virus primary infection as a result of the heat-killed DK128 pretreatment (Fig. 5a,c) were lethally challenged with heterosubtypic H1N1 (A/California/2009) pandemic virus at 4 weeks later (Fig. 5b). Naïve mice showed over 20% weight loss and 0% survival rates after infection with H1N1 pandemic virus (Fig. 5d). Mice that were protected against H3N2 A/Philippines/2/1982 virus primary infection as a result of heat-killed DK128 pretreatment displayed only transient 2–5% weight loss and 100% protection after secondary infection with heterosubtypic H1N1 virus (Fig. 5b,d).

To better understand the potential immune correlates conferring protection against secondary infection, an assay of hemagglutination inhibition (HI) was performed to determine cross protective HA antibodies. Consistent with IgG antibody levels (Fig. 4), sera from mice survived H3N2 primary infection regardless of LAB treatment showed high titers of HI activity against the homologous H3N2 virus (Supplementary Fig. 2a). Interestingly, sera from mice that were protected against H3N2 infection via prior LAB treatment showed substantial levels of HI activity against heterosubtypic H1N1 virus at a 2-fold higher level when compared to sera from mice survived H3N2 infection without prior LAB treatment (Supplementary Fig. 2b). Therefore, mice that were protected from primary infection (H3N2) via heat-killed DK128 pretreatment have acquired heterosubtypic immunity against secondary heterosubtypic infection (H1N1), most likely due to the elevated host immune responses during primary infection.

### C57BL/6 mice with heat-killed DK128 pretreatment acquire immunity against primary H1N1 and secondary rgH5N1 virus

**Figure 6:**
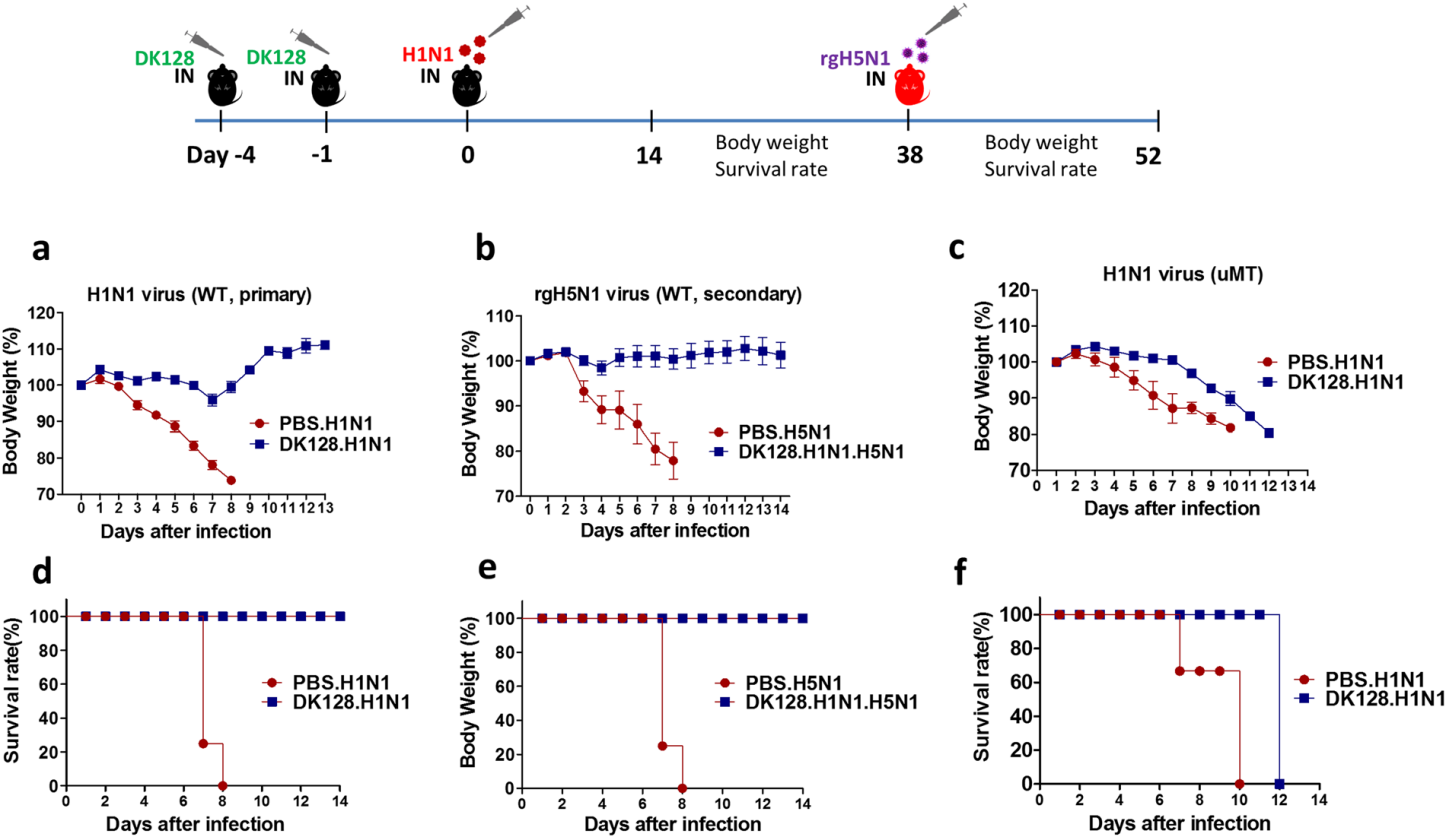
B cells are required to establish heat-killed DK128 mediated long-lasting immunity. Wilde type (C57BL/6) and B cell deficient (µMT) mice (n = 5/group) received intranasal pretreatment of heat-killed DK128 (10^9^ CFU/50 μl/mouse) at 4-day and 1-day prior to H1N1 (2 LD_50_, A/California/04/2009) virus for primary infection. Body weight change and survival rate were monitored for 14 days. The protected-mice against primary H1N1 virus via heat-killed DK128 pretreatment were then infected with secondary heterosubtypic virus rgH5N1 (5 LD_50_, reassortant with HA from A/Indonesia/05/2005), and then weight changes and survival rates were monitored for 14 days. (**a**) Body weight changes after primary infection of C57BL/6 mice with H1N1 virus. (**b**) Body weight changes after secondary infection of C57BL/6 mice with rgH5N1 virus. (**c**) Body weight changes in µMT mice with H1N1 virus infection. (**d**) Survival rates of C57BL/6 mice with H1N1 virus infection. (**e**) Survival rates of C57BL/6 mice with rgH5N1 secondary virus infection. (**f**) Survival rate of µMT mice with H1N1 primary virus infection. PBS.H1N1: H1N1 virus infection, DK128.H1N1: mice with heat-killed DK128 pretreatment prior to H1N1 virus infection. PBS.H5N1: rgH5N1 virus infection, DK128.H1N1.H5N1: The protected-mice against primary H1N1 virus via heat-killed DK128 pretreatment were then infected with secondary rgH5N1 heterosubtypic virus.

It is highly desirable if the protective effects of heat-killed LAB treatment would be expected in a strain non-specific pattern. We tested whether heat-killed DK128 pretreatment would mediate protection against a different subtype of influenza viruses in a different mouse strain. C57BL/6 mice (n = 5) were treated with heat-killed DK128 (1 × 10^9^ CFU) at days -4 and -1 prior to infection with H1N1 virus (A/California/2009). As we observed with BALB/c mice, heat-killed DK128 treated C57BL/6 mice showed protection against lethal H1N1 virus infection with minimal weight loss of approximately 5% (Fig. 6a,d). In contrast, the mock control group did not survive viral infection and all died by day 9 post infection (Fig. 6a,d). Additionally, we determined whether heat-killed DK128-treated C57BL/6 mice that were protected against H1N1 virus primary infection without weight loss would be also protected against secondary infection with rgH5N1 virus (Fig. 6b,e). C57BL/6 mice that were protected against primary H1N1 virus via heat-killed DK128 pretreatment were completely protected against lethal infection with the secondary rgH5N1 virus which was lethal for all naïve mice (Fig. 6b,e). These results suggest that heat-killed DK128 pretreatment can equip the mice with the capacity to confer protective immunity against a broader range of influenza A virus primary and secondary infections. Sera from mice survived H1N1 primary infection showed high titers of HI activity against the homologous H1N1 virus (Supplementary Fig. 2c) as well as significant levels of HI activity against heterosubtypic rgH5N1 virus, which are higher than those of naïve serum control (Supplementary Fig. 2b). Also, sera of H1N1 infection showed cross reactive binding antibodies against rgH5N1 virus (data not shown). Thus, cross-reactive immune responses developed during primary infection appear to be partially responsible for conferring protection against secondary infection.

### B cells are required for establishing protective immunity in mice with heat-killed DK128 treatment

We observed that heat-killed DK128 pretreated mice elicited earlier induction of IgG isotype-switched antibodies specific for the infecting virus (Fig. 4), indicating the important roles of B cells. Using a B-cell deficient (*µ*MT) mouse model, we further determined whether B-cells would play a critical role in establishing protective immunity against influenza virus infection via heat-killed DK128 pretreatments. B-cell deficient (*µ*MT) mice (n = 5) were treated with heat-killed DK128 (10^9^ CFU per mouse) at day 4 and day 1 prior to infection with H1N1 pandemic virus. Compared to the mock-treated *µ*MT mice after infection, heat-killed DK128 pretreated *µ*MT mice showed a delay of 3 to 5 days in displaying severe weight loss (Fig. 6c,f). Despite a prolonged delay in weight loss, the heat-killed DK128 treated *µ*MT mice were deemed to progressive morbidity and did not survive H1N1 virus infection. These results suggest that B cells are essential for establishing sustained long-lasting protection although a significant delay in weight loss might be due to innate immunity mediated by DK128 pretreatment.

### T cells are not required for protection against primary infection by heat-killed DK128 pretreatment but CD4 T cells partially contribute to preventing severe weight loss

**Figure 7:**
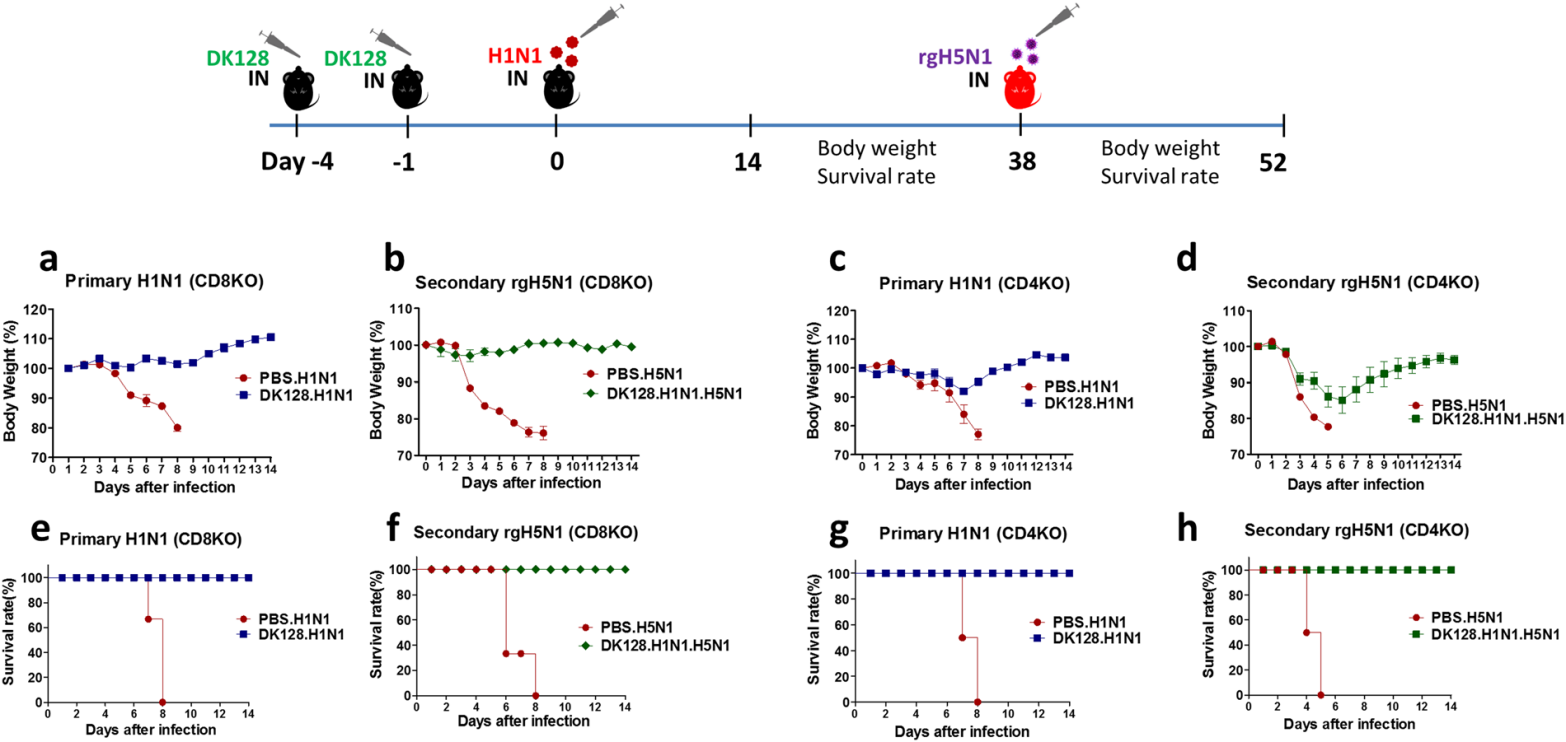
CD4 T cells contribute to preventing weight loss in heat-killed DK128 pretreated mice after primary or secondary infection. CD8-deficient (CD8KO) and CD4-deficient (CD4KO) mice (n = 5/group) received intranasal pretreatment of heat-killed DK128 (10^9^ CFU/50 μl/mouse) prior to H1N1 (2 LD_50_, A/California/04/2009) virus for primary infection, and then weight changes and survival rates were monitored for 14 days. The protected-mice against primary H1N1 virus via heat-killed DK128 pretreatment were then infected with secondary heterosubtypic virus rgH5N1 (5 LD_50_, A/Indonesia/05/2005), and then weight changes and survival rates were monitored for 14 days. (**a**) Body weight changes of CD8KO mice after primary infection with H1N1 virus. (**b**) Body weight changes of protected CD8KO mice against H1N1 virus after secondary infection with rgH5N1 virus. (**c**) Body weight changes of CD4KO mice after primary infection with H1N1 virus. (**d**) Body weight changes of CD4KO mice after secondary infection with rgH5N1 virus. (**e**) Survival rates of CD8KO mice after primary infection with H1N1 virus. (**f**) Survival rates of CD8KO mice after secondary infection with rgH5N1. (**g**) Survival rates of CD4KO mice after primary infection with H1N1 virus. (**h**) Survival rates of CD4KO mice after secondary infection with rgH5N1. PBS.H1N1: H1N1 virus infection, DK128.H1N1: mice with heat-killed DK128 pretreatment prior to H1N1 virus infection, PBS.H5N1: H5N1 virus infection, DK128.H1N1.H5N1: Protected mice against primary H1N1 virus via heat-killed DK128 pretreatment were exposed to secondary infection with rgH5N1.

In contrast to B cell-deficient mice, CD8 T cell-deficient (CD8KO) mice that received heat-killed DK128 pretreatment showed complete protection against H1N1 virus primary infection without weight loss (Fig. 7a,e). The CD4 T cell-deficient (CD4KO) mice that were pretreated with heat-killed DK128 were protected against H1N1 virus primary infection, displaying approximately 8% body weight loss and all CD4KO mice (100%) survived lethal infection (Fig. 7c,g). However, all mice died by day 9 post infection in the untreated mock control CD4KO and CD8KO groups (Fig. 7e,g).

Next, we determined the efficacy of subsequent protection against rgH5N1(A/Indonesia/05/2005) virus secondary infection in T cell-deficient mice that were well protected against H1N1 virus primary infection via heat-killed DK128 pretreatment (Fig. 7b,d). The CD8KO mice that were protected against H1N1 virus primary infection via heat-killed DK128 pretreatment without weight loss were well protected against rgH5N1 secondary virus, accompanying only a slight weight loss of 2–3% (Fig. 7b) under a condition with lethal infection (Fig. 7f). Whereas the CD4KO mice that survived H1N1 virus primary infection via killed-DK128 exhibited substantial weight loss of approximately 15–18% after secondary lethal infection with rgH5N1 virus under lethal infection (Fig. 7d,h). These results suggest that T cells are not needed for survival protection via heat-killed DK128 pretreatment against primary infection. However, CD4 T cells are required for establishing sufficient immunity to prevent severe weight loss during primary and secondary viral infections.

## Discussion

LAB as probiotics have been mostly studied using live bacteria that improve survival rates or partial protection against influenza virus. In the present study, we investigated the effects of heat-killed LAB DK128 on protective efficacy against heterologous influenza virus primary and secondary infections, and modulation of innate immune cells post infection. We found that mice that were pretreated with heat-killed DK128 were well protected against lethal influenza virus infection independent of strain specificity by lessening weight loss. The mice that were heat-killed DK128 treated and protected against influenza virus primary infection developed immunity contributing to heterosubtypic cross protection against subsequent secondary infection with heterosubtypic virus. In addition, B cells but not T cells were required for establishing prolonged immunity conferring protection via pretreatment with heat-killed DK128. These findings would significantly improve our understanding of mechanisms by which hosts can develop innate and adaptive immunity against lethal viral infections. It suggests a potential usage of LAB as antiviral probiotics.

In general, the hosts that experienced and survived severe disease due to natural viral infection are expected to develop strong immunity after recovery. In this study, heat-killed DK128 treated mice without experiencing severe illness during primary viral infection can develop strong immunity. It is significant that heat-killed DK128 pretreated and well protected mice against primary infection also developed cross protective immunity against secondary heterosubtypic virus. Rapid induction of IgG isotype switched antibodies might be a key mechanism for equipping the hosts with early immunity against pathogens. This study suggests that heat-killed DK128 treated mice can induce IgG1 and IgG2a antibodies at an earlier time and at higher levels while not exhibiting a sign of severe disease, such as body weight loss, compared with the mock-treated mice after lethal infection. Vaccination against seasonal influenza viruses usually induces strain-specific immune responses to vaccine strains^26^, while influenza virus infection induces broader cross-reactive and long-lasting immune responses when the host is re-infected by the same virus subtypes^27,28^. In this study, mice with pretreatment of heat-killed DK128 were protected against primary and heterosubtypic secondary influenza virus infections. Sera from mice protected against primary H1N1 virus or H3N2 virus via heat-killed LAB treatment exhibited high levels of homologous and heterosubtypic hemagglutination inhibiting activity during infection. Cross reactive antibodies elevated during primary infection are most likely contributing to conferring heterosubtypic protection during secondary lethal infections. In previous studies, mice that survived influenza virus infection and severe disease of weight loss are known to induce cross protective T cell responses, partially contributing to heterosubtypic protection during subsequent secondary lethal infections^29,30^. As expected, the survived control mice in the PBS.H3N2 group recovered from severe weight loss were protected during secondary virus infection (data not shown).

The importance of B cells was further evidenced by the finding that B cell-deficient mice were not able to establish long-lasting protection. A delay in weight loss was observed probably due to the induction of innate immunity as a result of pretreatment with heat-killed DK128. The CD8KO mice with heat-killed DK128 pretreatment did not show any weight loss, indicating that CD8 T cells might not play a critical role in conferring protection mediated by DK128 treatment. Meanwhile, CD4KO mice with heat-killed DK128 treatment showed a moderate weight loss (~5–8%) during primary infection and substantial weight loss of ~15–18% during secondary infection in contrast to wild type or CD8KO mice. Therefore, our study suggests that B cells are playing a critical role in establishing heat-killed DK128-mediated immunity; and CD4 T cells are partially involved in this protection probably helping the B cells in generating long lasting immunity.

Interestingly, we observed that alveolar macrophages were increased in the BALF and maintained in the lungs from the heat-killed DK128-treated mice whereas naïve mice showed a significantly lower level of macrophages in the lungs after viral infection. Better understanding of the innate immune cell mobilization during early time of infection provides a clue in the mechanism of protection. Alveolar macrophages are located at the interphase between air and lung tissue providing the first line of innate immune defense against influenza viruses^31^. Alveolar macrophages appear to play a critical role in conferring protection mediated by prior treatment with heat-killed LAB. Upon influenza virus infection, alveolar macrophages were depleted to lower levels correlating with disease and mortality^31^. Alveolar macrophages were reported to release inflammatory cytokines to control viral replication but did not support productive viral replications within the respiratory tract^32,33^. The increased populations of alveolar macrophages in BAL from heat-killed DK128 pretreated mice after H3N2 virus infection are likely to be partially responsible for conferring protection, which is further supported by macrophage depletion experiment results (Supplementary Fig. 1). Upon lethal infection with influenza virus, the clodronate-treated LAB mice displayed severe weight loss of over 20%. The importance of alveolar macrophages in LAB-mediated protection against influenza virus is consistent with a report that clodronate-mediated depletion of alveolar macrophages abolished the live LAB-mediated protection^20^. Other innate immune cells, such as monocytes and activated natural killer cells, were regulated to a lower level in the airways and lungs of the heat-killed DK128 treated mice, whereas the naïve mice recruited high levels of these inflammatory cells upon lethal infection. In addition, previous studies demonstrated that alveolar macrophages play a critical role in controlling lung viral loads and protection against influenza virus infections^34,35^. Heat-killed DK128 treatment likely modulates innate immune cells differentially depending on the cell types in lung microenvironment, which may contribute to controlling lung viral loads and protection during primary influenza virus infection.

Live LAB is more often studied probably because live *Lactobacillus* are expected to be more effective in improving survival rates of mice than dead bacteria as demonstrated by both oral and intranasal administration^24,36^. The efficacy of protection by LAB is variable in a wide range of improving survival protection to just delaying the death depending on the strains of choice^3,17,19–22,24,37^. In terms of safety concerns, heat-killed LAB would be a desirable choice for developing safer probiotics. Dendritic cells are professional antigen presenting innate immune cells playing a critical role in generating cell-mediated immunity. Induction of proinflammatory cytokines is considered a possible mechanism of LAB-mediated antiviral effects^38,39^ although high levels of inflammatory cytokines caused inflammatory pulmonary disease in mice that were lethally infected with pathogenic influenza viruses^40,41^. The LAB experimental protocols are various and pretreatment periods are from few days to several weeks via either intranasal or oral routes^14,16,20,37,42,43^. In this study, we found that mice that were pretreated with heat-killed DK128 were protected against lethal infection and protected against severe weight loss. Even a single treatment resulted in effective protection against lethal infection as well as preventing weight loss (data not shown). These results are notable that DK128 strain as a novel strain of *Lactobacillus* tested in this study might have potent protective effects on equipping the hosts with immunity against influenza virus in a form of dead LAB.

A possible mechanism of heat-killed LAB DK128 antiviral effects is that innate immune cells and cytokines induced by heat-killed DK128 pretreatment control or delay viral replication to a lower level which does not cause disease. Even the B cell deficient mice with heat-killed DK128 pretreatment showed a delay of 3–5 days before displaying a sign of severe weight loss and mortality. Thus, the immediate control of viral replication by protective innate immunity might provide sufficient time for hosts to establish heterosubtypic cross protective immunity via B cells. This idea is further supported by the important roles of CD4 T cells in establishing heterosubtypic immunity during primary and secondary infection probably by providing the help to B cells. Also, moderate innate inflammation induced by heat-killed DK128 pretreatment may provide favorable microenvironment to expedite the earlier induction of adaptive immunity. Further studies will be required in testing this hypothesis.

Oral administration of live LAB was also reported to provide beneficial effects on mitigating severe disease due to influenza virus infection in mice^14^. It remains to be determined whether heat-killed LAB would confer similar protection against influenza virus via daily diet. Air transmission of influenza virus spreads rapidly among susceptible humans as exampled by the 2009 H1N1 pandemic^44^. FluMist® is licensed influenza nasal spray vaccine formulations. Our current study provides a piece of evidence that heat-killed LAB could potentially be administered via a nasal spray form as a prophylactic drug against non-specific influenza virus infections.

## Methods

### **Preparation of*Lactobacillus casei*DK128 and virus**

*Lactobacillus casei* (DK128) obtained from Dankook University in South Korea were isolated from fermented Korean vegetable food “Kimchi” as described^25^. MRS (de Man, Rogosa and Sharpe) broth (Becton Dickinson, Sparks, MD) was used to culture DK128 for 17–24 hr at 37 °C. LAB DK128 was harvested by spinning down at 8,000 rpm at 4 °C for 5 minutes, washed twice with phosphate-buffered saline (PBS), and then resuspended in PBS. Heat-killed DK128 was prepared by heating DK128 at 95 °C for 30 minutes and inactivation was confirmed by the absence of colony formation on the MRS agar plate. The reverse genetic reassortant rgH5N1 virus contains HA from A/Indonesia/5/2005 (H5N1), and the remaining 7 backbone genes from A/Puerto Rico/8/34^45,46^. Influenza viruses A/Philippines/2/1982 (H3N2), A/California/04/2009 (H1N1), and rgH5N1 were grown in embryonated chicken eggs and stored in −80 °C as described^20,45,47^. Heat-killed LAB would not cause disease in humans and belong to biosafety level 1.

### Treatment of mice with LAB DK128 and infection with influenza viruses

Female BALB/c mice (6–8 weeks old, Harlan Laboratories) and C57BL/6 mice (Jackson Laboratory) were used (n = 5 each group) in this study. For intranasal administration of heat-killed LAB DK128, mice were anesthetized with isoflurane using an oxygen controlled machine (Baxter, Deerfield, IL) and then administered heat-killed LAB DK128 (10^7^ to 10^9^ CFU, colony forming units, per mouse). For primary virus infection, naïve or LAB treated mice were infected with A/Philippines/82 H3N2 virus, H1N1 pandemic virus (A/California/2009), or rgH5N1(A/Indonesia/05/2005) virus at a lethal dose (2 x LD_50,_ 50% lethal dose). A control group was infected with the same dose of H3N2, H1N1, or rgH5N1 virus without LAB DK128 probiotic treatment. For mutant mice, B cell-deficient mice (B6.129S2-*Ighm* ^*tm1Cgn*^/J), CD4-deficient mice (B6.129S2-Cd4tm1Mak/J), and CD8-deficient mice (B6.129S2-Cd8atm1Mak/J) were purchased from Jackson Laboratory (Bar Harbor, Maine). These mutant mice were intranasally pre-treated with heat-killed LAB DK128 (10^9^ CFU per mouse) prior to lethal infection with influenza virus. All infected animals were daily monitored, and their weight and survival rates were recorded by following humanely handling animal procedures. Animal experiments presented in this study were approved by the Georgia State University (GSU) Institutional Animal Care and Use protocol (IACUC) review boards. All animal experiments and husbandry have been carried out under the guidelines of the GSU IACUC. All influenza virus infection experiments were carried out in the biosafety level 2 animal facility.

### Lung and bronchoalveolar lavage fluid (BALF) cells and sample preparations

Mice of each group were euthanized at the indicated date post infection. The lungs were removed aseptically and homogenized in Roswell Park Memorial Institute (RPMI) medium 1640 (Fisher Scientific, Corning cellgro, USA) using 0.4 *um* cell strainer. The homogenates were then centrifuged at 1,700 rpm for 10 minutes. The supernatants were stored at −80 °C for other assays. The cell pellet was resuspended in 44% then carefully layered on to 66% Percoll and centrifuged at 2,700 rpm for 15 minutes. After the centrifugation, two layers were formed and the interspace between the two Percoll concentrations were collected using the sterilized transfer pipet. The layers were then washed in cold PBS and ready for counting cells to check their viability assessed by Trypan blue (Sigma). The bronchoalveolar lavage (BAL) fluid was collected from the trachea using 18-gauge Excel Safelet Catheter. A small incision in the trachea was made to allow passage of 18-gauge lavage tube into trachea. 800 ul of cold PBS was injected into lung through the incision for two times and the BALF was collected in 5 ml tube. The BALF was then centrifuged at 1,700 rpm for 5 minutes. The supernatant was stored at −80 °C for cytokine ELISA and the cell pellet was lysed using red blood cell lysis buffer and resuspended in PBS to stain for FACS analysis.

### Flow cytometry analysis of innate immune cells

The prepared lung and BAL cells were stained with different surface markers. The cell surface markers for flow cytometry analysis included CD11c^+^CD11b^−^F4/80^+^MHCII^hi^ (alveolar macrophage), F4/80^−^DX5^+^CD69^+^ (natural killer Cells), F4/80^−^DX5^+^CD69^+^(activated natural killer cells), and CD11c^−^CD11b^+^siglecF^−^Ly6c^hi^F4/80^+^ (monocytes) as described^48^. Stained cell samples were performed by Becton-Dickenson LSR flow cytometer and data analysis by Flowjo Software Program (Tree Star Inc.).

### Antibody and cytokine ELISA

To measure antibody responses in serum samples, the antigen of inactivated virus (H3N2 A/Philippines/82, H1N1 A/California/09, rgH5N1) was coated onto the 96-well ELISA plates for overnight at 4 °C and washed with phosphate buffered saline Tween 20 (0.05%). Blocking was done with 3% bovine serum albumin containing PBS (3% BSA) at room temperature (RT) for 1.5 hr. The serially diluted serum samples were added to each well and incubated at RT for 1.5 hr. After washing, secondary antibodies were added and incubated at RT for 1hr. Then tetramethylbenzidine (TMB) substrate was added to detect horseradish peroxidase (HRP) enzyme conjugate activity, which was measured by optical density at 450 nm. Levels of cytokines in BALF and lung were determined according to Read-Set-Go IL-6 and TNF-α cytokine kits by ELISA as described (eBioscience, San Diego, CA) as previously described^20,48^.

### Lung viral titers

Virus titers in the lung homogenates were determined by infectivity doses of serially diluted samples in embryonated chicken eggs as previously described^45,46^.

### Statistical analysis

All data were statistically analyzed using a GraphPad Prism version 5.01 software (GraphPad Software Inc, La Jolla, CA, USA). The statistical significances of two different groups were stated using two-tailed student’s paired *t* test and one-way ANOVA. A *P* value < 0.05 was considered to be significant.

## Electronic supplementary material

Supplementary information
