# Supplementary material for: Heat-killed Lactobacillus casei confers broad protection against influenza A virus primary infection and develops heterosubtypic immunity against future secondary infection
Source: Sci Rep. 2017 Dec 12;7:17360. doi: 10.1038/s41598-017-17487-8 (PMC5727132; doi:10.1038/s41598-017-17487-8)
Supplement: Supplementary file 1 — Supplementary information [file 41598_2017_17487_MOESM1_ESM.pdf]

Heat-killed *Lactobacillus casei* confers broad protection against influenza A virus primary infection and develops heterosubtypic immunity against future secondary infection

Yu-Jin Jung, Young-Tae Lee, Vu Le Ngo, Young-Hee Cho, Eun-Ju Ko, Sung-Moon Hong,
Ki-Hye Kim, Ji-Hun Jang, Joon-Suk Oh, Min-Kyung Park,
Cheol-Hyun Kim, Jun Sun, Sang-Moo Kang

Supplementary Fig. 1

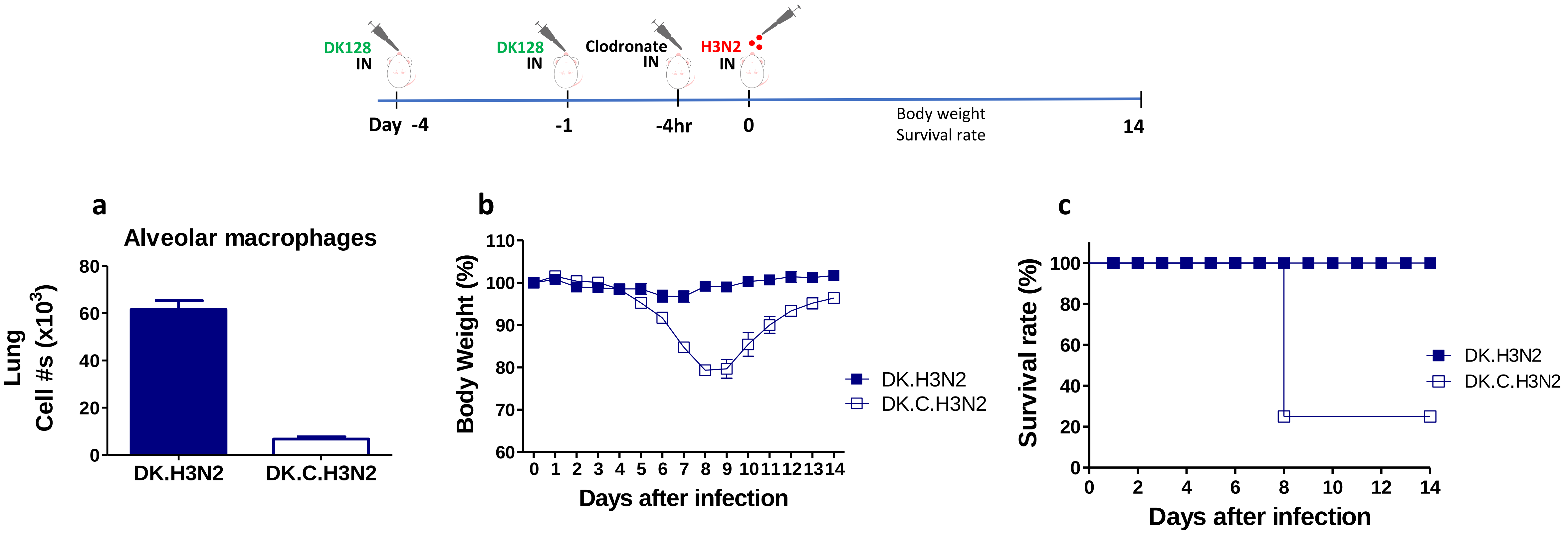

Supplementary Figure 1. Effects of alveolar macrophage depletion on conferring protection against virus infection.

BALB/c mice (n=5/group) received intranasal pretreatment of heat-killed DK128 (10^9 CFU/50µl/mouse) at -4 day and -1 day prior to H3N2 ($1.5LD_{50}$, A/Philippines/2/1982) virus infection. (**a**) Alveolar macrophages ($CD11c^+CD11b^-F4/80^+$) in lung. BALB/c mice (n=5/group) with heat-killed DK128 (10^9 CFU/50µl/mouse) were treated with clodronate-liposome or PBS intranasally before H3N2 ($1.5LD_{50}$, A/Philippines/2/1982) virus infection. At 7 days after virus infection, lungs were collected and alveolar macrophages were determined. (**b**) Body weight changes of BALB/c mice with H3N2 virus infection. (**c**) Survival rates of BALB/c mice with H3N2 virus infection. DK128.H3N2: mice with heat-killed DK128 pretreatment prior to H3N2 virus infection, DK128.C.H3N2: mice with heat-killed DK128 then clodronate-liposome treatment prior to H3N2 virus infection.

Supplementary Fig. 2

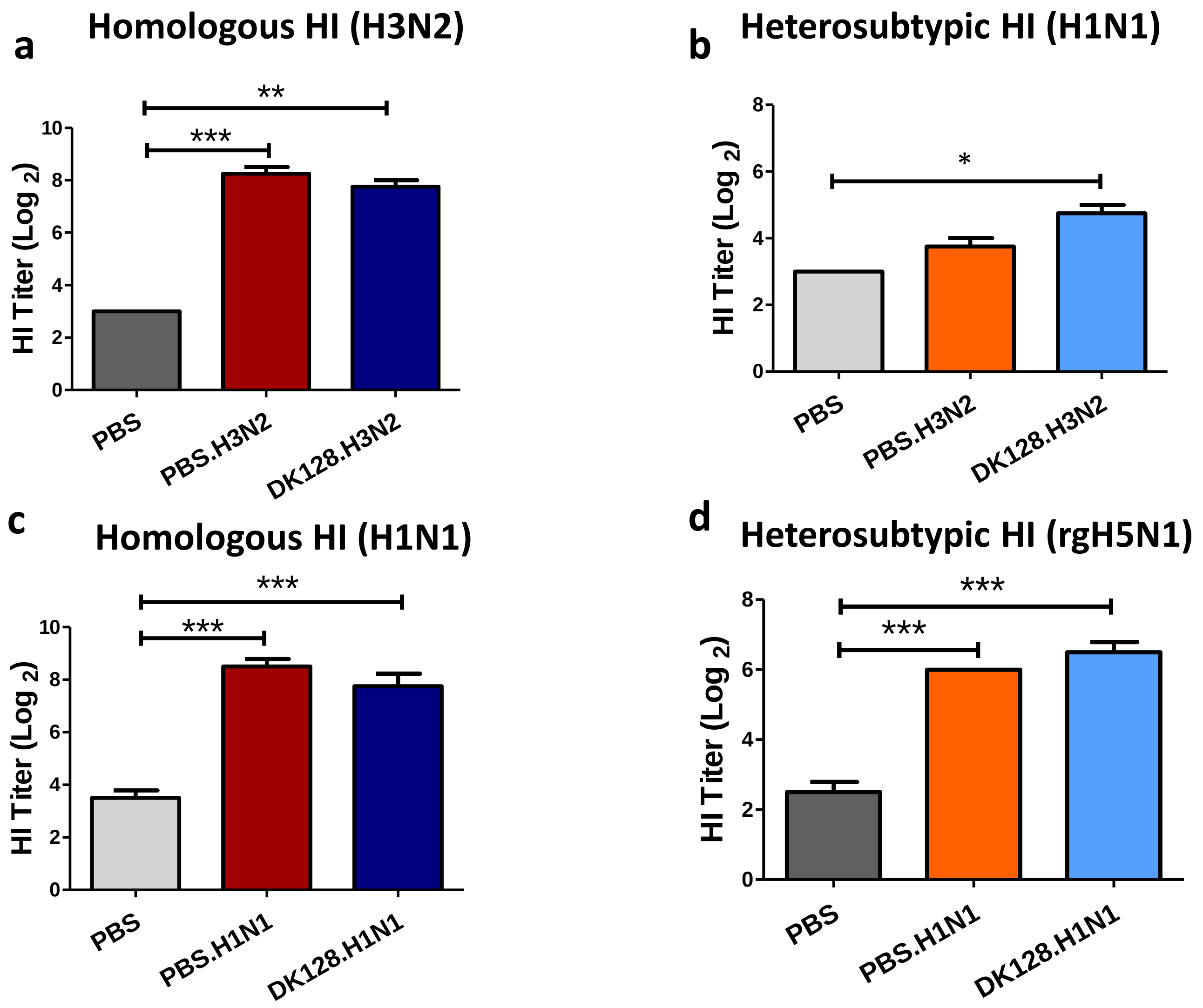

Supplementary Figure 2. Homologous and heterosubtypic hemagglutination inhibition (HI) activity in sera from mice with primary virus infection.

(**a**) Homologous HI activity of H3N2 virus infection sera against H3N2 virus. (**b**) Heterosubtypic HI activity of H3N2 virus infection sera against H1N1 virus. (**a-b**) Sera collected day 14 post infection from BALB/c mice (n=5/group) that were intranasally pretreated with heat-killed DK128 and then infected with H3N2 virus. (**c**) Homologous HI activity of H1N1 virus infection sera against H1N1 virus. (**d**) Heterosubtypic HI activity of H1N1 virus infection sera against rgH5N1 virus. (**c-d**) Sera collected day 14 post infection from C5BL/6 mice (n=5/group) that were intranasally pretreated with heat-killed DK128 prior to H1N1 virus infection. PBS: mock-treated mouse sera, PBS.H3N2: Sera of mock-treated BALB/c mice infected with H3N2 influenza virus. PBS.H1N1: Sera of mock-treated C5BL/6 mice infected with H1N1 influenza virus. DK128.H3N2: Sera of BALB/c mice with heat-killed DK128 pretreatment prior to H3N2 virus infection. DK128.H1N1: Sera of C5BL/6 mice with heat-killed DK128 pretreatment prior to H1N1 virus infection. HI titer in serum was described as mean ±SEM. *, **, and *** denote $p<0.05$, 0.1, and 0.001 respectively by One-way ANOVA and Tukey's multiple comparison test.

Material and methods

Hemagglutination inhibition (HI) assays

As an indicative measure of protective antibody responses, HI titers against homologous and heterologous influenza viruses in serum samples from infected mice were determined as described [1-3]. In brief, the immune sera collected from mice that survived virus infection were incubated with receptor destroying enzymes (RDE, Sigma) at 37 ◦C overnight. Then, serum samples were incubated at 56 ◦C for 30 minutes to inactivate RDE and complement activity. Inactivated sera were serially diluted in 96-well V-bottom plates, 4 hemagglutination units (HAU) of viruses were added and incubated at room temperature for 30 minutes. After 30 minutes incubation, 0.5 % chicken red blood cell solution (Lampire Biological) was added to the serum-virus mixture. The hemagglutination inhibition as indicated by forming a button was observed after 40 minutes incubation at room temperature. Duplicate measurements were assessed.

Alveolar macrophage depletion by clodronate-liposome treatment

To deplete alveolar macrophages, BALB/c mice were intranasally treated with 100ul of clodronate-liposome (ClodronateLiposome) after the mice were anesthetized with isoflurane as described [4,5]. Mice were treated with clodronate-liposome 4 hours prior to virus infection and euthanized at 7 days after virus infection to confirm the depletion of alveolar macrophages using flow cytometry.

References

1 Lee, Y. T. *et al.* Innate and adaptive cellular phenotypes contributing to pulmonary disease in mice after respiratory syncytial virus immunization and infection. *Virology* **485**, 36-46, doi:10.1016/j.virol.2015.07.001 (2015).

2 Kim, M. C. *et al.* Supplementation of influenza split vaccines with conserved M2 ectodomains overcomes strain specificity and provides long-term cross protection. *Mol Ther* **22**, 1364-1374, doi:10.1038/mt.2014.33 (2014).

3 Ko, E. J. *et al.* Effects of MF59 Adjuvant on Induction of Isotype-Switched IgG Antibodies and Protection after Immunization with T-Dependent Influenza Virus Vaccine in the Absence of CD4+ T Cells. *J Virol* **90**, 6976-6988, doi:10.1128/JVI.00339-16 (2016).

4 Tate, M. D., Schilter, H. C., Brooks, A. G. & Reading, P. C. Responses of mouse airway epithelial cells and alveolar macrophages to virulent and avirulent strains of influenza A virus. *Viral immunology* **24**, 77-88, doi:10.1089/vim.2010.0118 (2011).

5 Lee, Y. T. *et al.* Respiratory syncytial virus-like nanoparticle vaccination induces long-term protection without pulmonary disease by modulating cytokines and T-cells partially through alveolar macrophages. *Int J Nanomedicine* **10**, 4491-4505, doi:10.2147/IJN.S83493 (2015).
